# Orofacial Drinking Tremor: A Case Series and Literature Review

**DOI:** 10.1002/mdc3.70621

**Published:** 2026-04-10

**Authors:** Daniele Birreci, Luca Angelini, Simone Aloisio, Anna S. Grandolfo, Sara Cirinei, Adriana Martini, Martina De Riggi, Matteo Bologna

**Affiliations:** ^1^ Department of Human Neurosciences Sapienza University of Rome Rome Italy; ^2^ IRCCS Neuromed Pozzilli Italy; ^3^ Department of Translational and Precision Medicine Sapienza University of Rome Rome Italy

**Keywords:** drinking tremor, orofacial tremor, position‐specific, task‐specific, unclassified tremor

## Abstract

**Background:**

Task‐specific orofacial tremor is a rare condition in which rhythmic oscillations of orofacial muscles occur during specific actions. Drinking tremor represents a recurrent pattern in isolated reports, although its phenomenology and underlying mechanisms remain incompletely defined.

**Cases:**

We report three cases of orofacial drinking tremor, each showing a different pattern. Differences involved the distribution of orofacial muscle activity and the presence of additional signs, including tremor in other body regions or subtle dystonic postures. Variability in task dependence, posture sensitivity, and sensory modulation contributed to the heterogeneity of these presentations.

**Literature Review:**

Published reports describe a spectrum of orofacial drinking tremors, ranging from strictly task‐specific patterns to mixed task‐ and position‐dependent presentations. Some patients exhibit subtle dystonic features, whereas others show no clear additional neurological signs. Electrophysiology typically demonstrates regular 5–8 Hz rhythmic discharges in the orofacial muscles. Systemic pharmacological therapies offer limited benefit, whereas botulinum toxin remains the most effective option.

**Conclusions:**

Current evidence suggests that orofacial drinking tremor does not represent a single clinical entity. An approach integrating detailed phenomenological characterization with plausible pathophysiological mechanisms may improve diagnostic interpretation and guide individualized management strategies.

Task‐specific orofacial tremor is a rare tremor syndrome characterized by involuntary, rhythmic oscillations of the orofacial muscles that occur exclusively during a particular task.^1^, ^2^, ^3^ Among its task‐dependent variants, drinking tremor has been described in some reports as a recurrent clinical presentation. However, in most cases the tremor is not strictly task‐exclusive, as it may also appear during specific mouth postures and can be alleviated by compensatory maneuvers such as drinking through a straw.^4^, ^5^, ^6^, ^7^, ^8^, ^9^, ^10^, ^11^ The etiology is often idiopathic and the condition can be disabling and socially stigmatizing. Clinical evaluation should include questions regarding the use of wind instruments and prior dental procedures, as these have been described as potential risk factors.^4^, ^7^, ^12^ Given the focal nature of this tremor, botulinum toxin (BoNT) is generally the most effective therapeutic option, whereas systemic medications show poor efficacy. Although isolated reports exist, the phenomenology of drinking tremor remains poorly defined. Here, we present three cases of orofacial drinking tremor referred to the outpatient clinic at Sapienza University of Rome, all of whom underwent clinical and kinematic assessments,^13^ with all recordings obtained outside the BoNT effect window. Each case exhibits slightly differing patterns with variable involvement of orofacial muscles, as well as tremor in other body segments or additional signs of uncertain significance, such as dystonic features and rest tremor.

## Case Series

### Case 1

A 73‐year‐old woman presented with a three‐year history of slowly progressive upper limb tremor, initially right‐sided and subsequently bilateral. The tremor was mainly action‐related, occasionally perceived at rest, without clear impact on daily activities. During the last year, she noticed the appearance of a jaw tremor, occurring almost exclusively while drinking from a glass, which had progressively worsened. She reported no history of wind instrument use or recent dental procedures. Her past medical history included hypercholesterolemia and thrombocytosis under hematologic follow‐up. No family history of tremor was reported.

**Figure 1:**
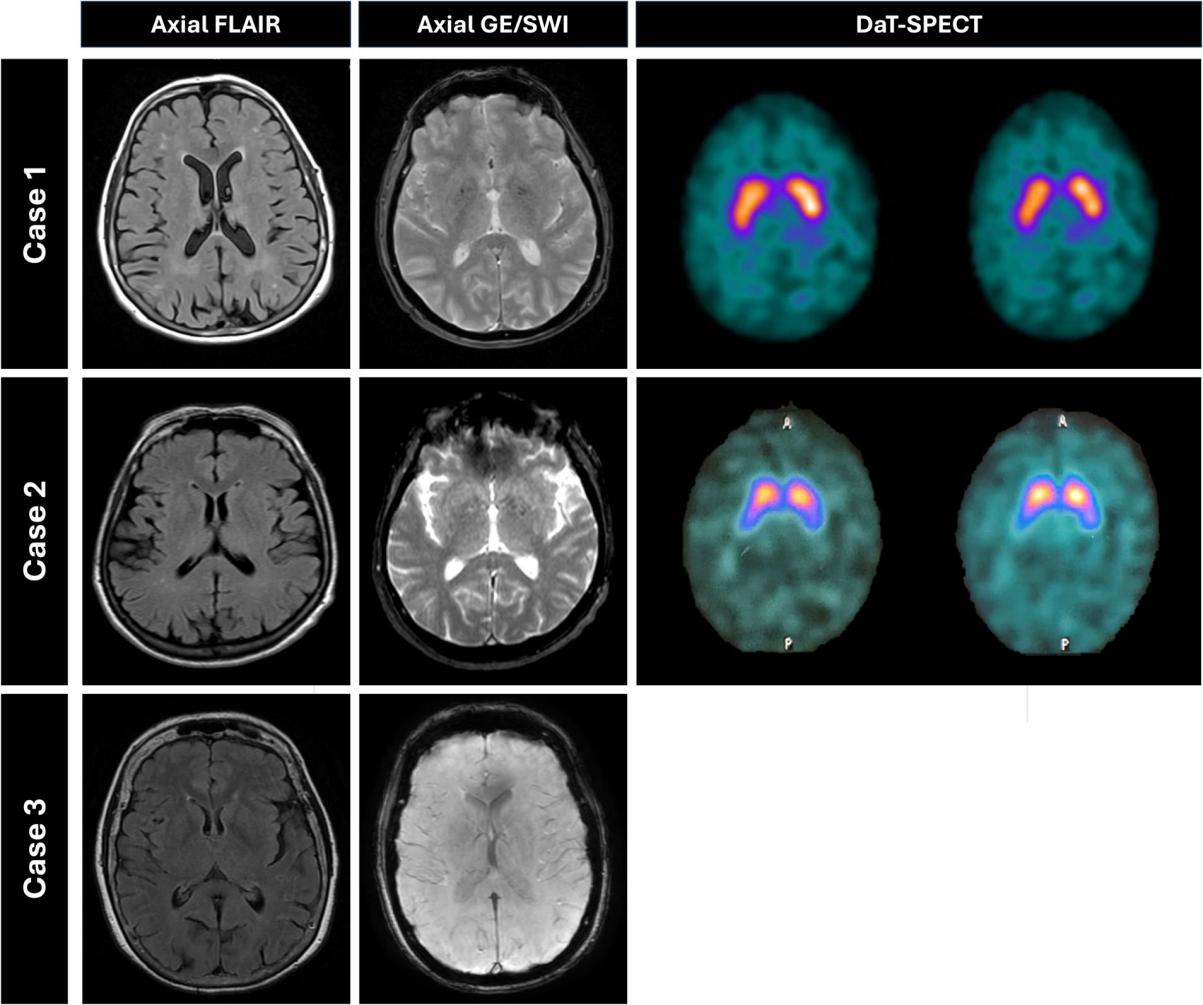
Brain MRI and DaT‐SPECT data in the three cases. Axial fluid‐attenuated inversion recovery (FLAIR) sequences, revealing mild white‐matter hyperintensities without focal abnormalities in all three cases. Gradient‐echo/susceptibility‐weighted imaging (GE/SWI) sequences, revealing minimal basal ganglia iron deposition in cases 1 and 2. DaT‐SPECT images demonstrating a normal presynaptic dopaminergic uptake pattern with no evidence of nigrostriatal denervation.

At the time of evaluation, she was taking clonazepam 0.6 mg/day (0.3 mg twice daily), which caused excessive daytime sleepiness and was therefore discontinued. Previous therapeutic trials prescribed at another center prior to our evaluation included pramipexole extended release 0.52 mg/day (once daily), without significant benefit, and propranolol 20 mg/day (10 mg twice daily), discontinued because of mild bradycardia and limited efficacy. A levodopa trial was not performed due to the absence of clear clinical progression over time, the lack of response to prior dopaminergic therapy, and the mild functional impact of symptoms. She is currently experiencing some clinical benefit from BoNT injections into the masseters and mentalis muscles (5 UI per side for each muscle; Table S1). Brain MRI showed very mild chronic small‐vessel ischemic leukoencephalopathy. DaT‐SPECT demonstrated a normal nigrostriatal dopaminergic uptake pattern (Fig. 1).

**Figure 2:**
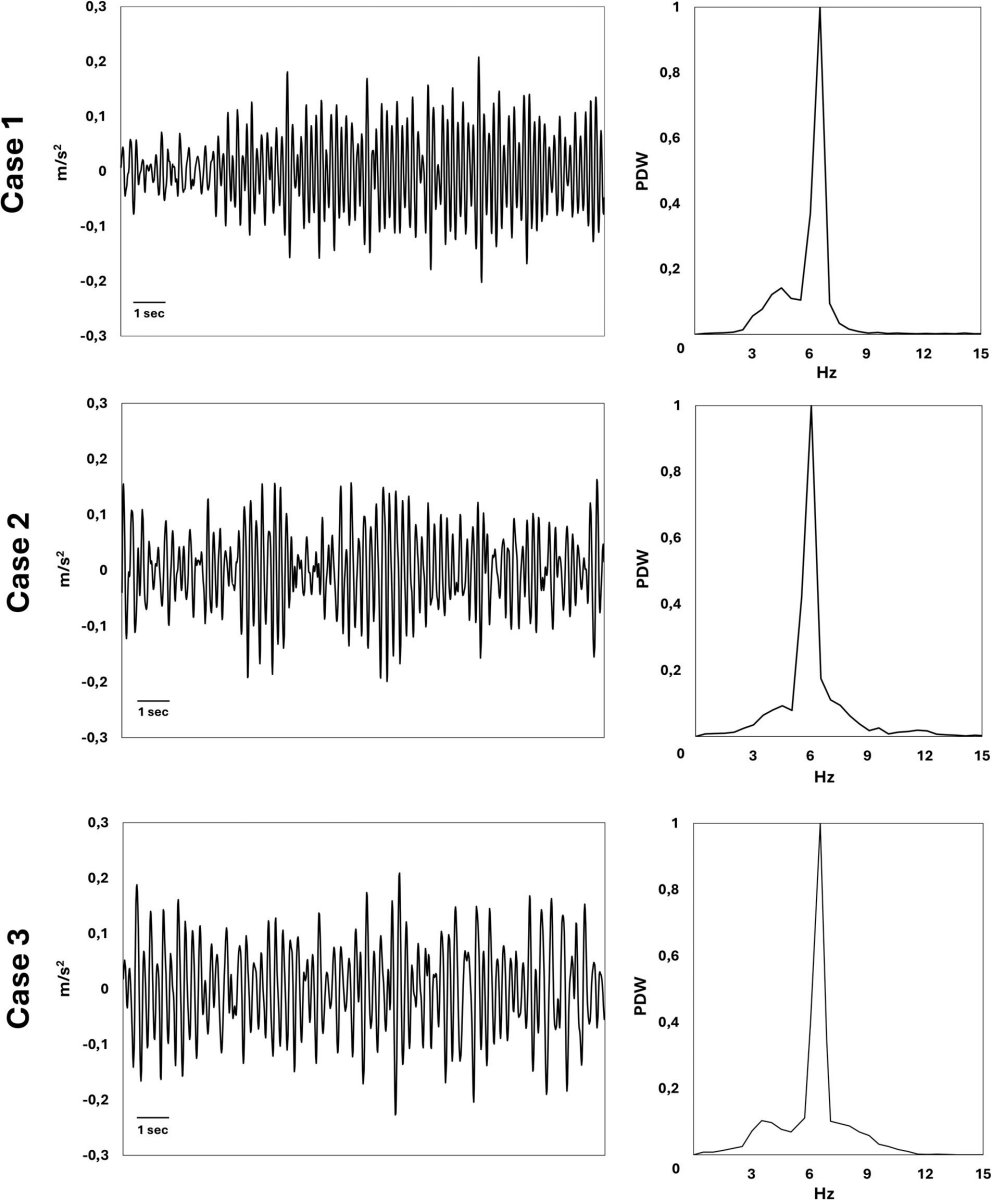
Kinematic tremor traces and frequency spectra across the three cases. Left panels: Kinematic traces of tremor amplitude recorded during the drinking task, expressed as acceleration signal (m/s^2^). RMS of these traces reflects the magnitude of oscillatory acceleration over time and provides a quantitative estimate of tremor amplitude. Right panels: Frequency spectra expressed as power density waveform (PDW), corresponding to the normalized power spectral density of the tremor signal. The PDW peak indicates the dominant tremor frequency (Hz).

Neurological examination revealed a tremor involving the jaw, as well as the perioral and perinasal muscles, appearing predominantly during drinking. The tremor diminished when mimicking drinking with an empty glass and disappeared when she drank through a straw. It was absent at rest, when she held water in the mouth, during mastication, and during tasks such as whistling or mimicking a kiss. An intermittent bilateral upper limb rest tremor was observed, slightly more prominent on the right. A very mild postural and kinetic upper limb tremor was present, without a re‐emergent component. Finger tapping revealed mild right‐sided bradykinesia without rigidity. Gait assessment demonstrated reduced right arm swing with a walking tremor on the same side (Video 1). Kinematic analysis of drinking‐related orofacial tremor revealed a peak frequency of 6.5 Hz and a mean amplitude of 0.043 m/s^2^ root mean square (RMS) (Fig. 2).

**Video 1:** Neurological examination in Case 1, including drinking from a glass, holding water in the mouth, mimicking drinking with an empty glass, drinking through a straw, and eating. Additional orofacial assessments comprise observation of the mouth at rest and slightly open, whistling, and smiling. Upper‐limb evaluation is performed at rest, during three maintained postures, with the finger‐to‐nose test and finger tapping, followed by gait examination.

### Case 2

A 70‐year‐old woman presented with an eight‐year history of a mild jaw tremor that markedly worsened while drinking liquids, interfering with daily activities. She reported minimal upper limb action tremor, which had remained stable over time. She reported no history of wind instrument use or recent dental procedures. Her past medical history included arterial hypertension, dyslipidemia, and an ischemic stroke in 1976, likely embolic in origin, which occurred during pregnancy and left her with subtle clumsiness in the right upper limb. Her mother had a history of tremor affecting the upper limbs and head, with onset around the age of 80.

At the time of evaluation, she was taking propranolol 40 mg/day (once daily) and clonazepam 0.5 mg/day (at bedtime), with minimal clinical benefit. She is currently experiencing meaningful improvement with BoNT injections to the mentalis muscles (7.5 UI per side; Table S1). Brain MRI showed mild, scattered white‐matter hyperintensities. DaT‐SPECT demonstrated a normal nigrostriatal dopaminergic uptake pattern (Fig. 1).

Neurological examination revealed a tremor involving the jaw and lips, predominantly triggered by drinking. The tremor disappeared when the patient mimicked drinking from an empty glass or used a straw. A mild jaw tremor was also present at rest with the mouth closed, worsening when the mouth was half open, but disappearing during smiling or whistling. A minimal postural tremor was observed in the right upper limb, while no rest tremor was detected. Finger tapping did not show definite bradykinesia. Gait was regular and stable, with mild left shoulder elevation (Video 2). Kinematic analysis of the orofacial drinking tremor revealed a peak frequency of 6.0 Hz and a mean amplitude of 0.051 m/s^2^ RMS (Fig. 2).

**Video 2:** Neurological examination in Case 2, including drinking from a glass, holding water in the mouth, mimicking drinking with an empty glass, drinking through a straw, and eating. Additional orofacial assessments comprise observation of the mouth at rest and slightly open, whistling, and smiling. Upper‐limb evaluation is performed at rest, during three maintained postures, with the finger‐to‐nose test and finger tapping, followed by gait examination.

### Case 3

A 73‐year‐old woman presented with a three‐year history of chin and jaw tremor, most pronounced while drinking liquids but intermittently present at rest, with mild progression over time. She also reported a minimal postural and kinetic tremor of the upper limbs. No history of wind instrument use or recent dental procedures was reported. Her past medical history included arterial hypertension and dyslipidemia. Her mother had a history of tremor affecting the chin and upper limbs, with late‐life onset.

At the time of evaluation, the patient was taking escitalopram 10 mg daily for a reactive depressive episode, introduced approximately one year earlier, without any reported worsening of the tremor. No tremor‐specific medications had been previously tried. She is currently experiencing clinical benefit from BoNT injections to the masseters and mentalis muscles (5 UI per side for each muscle; Table S1). Brain MRI showed mild, scattered white matter hyperintensities (Fig. 1).

Neurological examination revealed a jaw and chin tremor predominantly during drinking, persisting with reduced amplitude when using an empty glass. The tremor was barely visible with the mouth half open and absent at rest. Minimal kinetic tremor of the upper limbs was present, without rest tremor. Finger tapping showed no definite bradykinesia, though mirror movements appeared in the right hand during left‐hand tapping. Subtle left torticollis with right laterocollis, along with mild left shoulder elevation, were observed. Gait was normal (Video 3). Kinematic analysis of the orofacial drinking tremor revealed a peak frequency of 6.5 Hz and a mean amplitude of 0.046 m/s^2^ RMS (Fig. 2).

**Video 3:** Neurological examination in Case 3, including drinking from a glass, holding water in the mouth, mimicking drinking with an empty glass, drinking through a straw, and eating. Additional orofacial assessments comprise observation of the mouth at rest and slightly open, whistling, and smiling. Upper‐limb evaluation is performed at rest, during three maintained postures, with the finger‐to‐nose test and finger tapping, followed by gait examination.

## Literature Review

A focused literature search was conducted in PubMed without time or language restrictions and was executed on November 12, 2025. The search strategy was based on relevant free‐text terms related to task‐specific orofacial tremor, including “task‐specific tremor,” “drinking tremor,” “orolingual tremor,” “orofacial tremor,” and “jaw tremor.” Reference lists of relevant articles were also screened to identify additional reports. Titles and abstracts were reviewed, and articles were included if they described patients with tremor predominantly or exclusively triggered by drinking. Articles were excluded if drinking was not a primary triggering condition. All included studies were single case reports, yielding 10 cases summarized in Table 1.

**TABLE 1:** Summary of reported cases of drinking‐related tremor in the reviewed literature

Study	Age (y)	Sex	Age at onset (y)	Family history	Task‐dependent phenomenology	Aggravating or relieving factors	Electrophysiological findings	Brain MRI	Treatment
Miles et al, 1997	61	F	59	No	Jaw tremor occurring while drinking and during jaw closure (teeth contact) or with jaw protrusion/rightward rotation; absent at rest, during speech, jaw clenching, or wide mouth opening	Aggravated by bruxism; no improvement with alcohol	5–6 Hz rhythmic activity in digastric muscles	Normal	NA
Tarsy and Ro, 2006	66	F	62	No	Jaw tremor occurring while drinking, speaking, and with the mouth partially open; absent at rest, with the mouth closed or wide open, and during eating	Aggravated by stress, improvement with alcohol	5 Hz rhythmic activity in bilateral masseter and digastric muscles; no co‐contraction of antagonist muscles	NA	BoNT to digastric and masseter muscles with clinical benefit
O’Gorman et al, 2014	49	F	49	Yes	Jaw and lip tremor occurring while drinking; absent at rest, while drinking with a straw, holding liquids in the mouth, eating, or speaking	Improvement with alcohol	7.5–8 Hz rhythmic activity in bilateral upper orbicularis oris muscles	Minimal leukoaraiosis	Declined treatment
Macerollo et al, 2016	58	F	56	No	Lip tremor occurring only while drinking from a cup; absent at rest, while speaking or eating	No improvement with alcohol	8 Hz rhythmic activity in bilateral upper and lower orbicularis oris muscles	Normal	No efficacy from propranolol, trihexyphenidyl, primidone, or BoNT
Carpentier et al, 2016	69	F	68	No	Jaw tremor occurring only while drinking from a cup; absent at rest, while speaking or eating	Onset after bilateral gingival grafting; touching upper lip with liquid container alleviated tremor; no improvement with alcohol	NA	Normal	Declined treatment
Stampanoni Bassi et al, 2019	74	M	69	No	Chin and lip tremor occurring only while drinking from a cup; absent at rest, while drinking with a straw, holding liquids in the mouth, speaking or eating	No improvement with alcohol	7.5–8 Hz rhythmic activity in upper orbicularis oris and digastric muscles; subclinical dysphagia at EPSS	Normal	No efficacy from propranolol, declined BoNT
Lau et al, 2023	75	F	72	No	Jaw tremor occurring while drinking from a cup or a bottle, reduced while drinking with a straw; absent at rest, while speaking or eating	NA	NA	Mild cerebral atrophy (age‐related)	No efficacy from trihexyphenidyl or propranolol, declined BoNT
Benedek et al, 2023	63	F	63 (sudden onset)	No	Oro‐mandibular and left upper‐limb tremor occurring while drinking from a glass or a bottle, or when the cup touches the lip; absent at rest, while drinking with a straw, speaking or eating	Aggravated by stress	Irregular 4–7 Hz bursts in orbicularis oris muscles	Normal	BoNT to orbicularis oris and digastric muscles with clinical benefit
Procaci et al, 2025	80	F	75	NA	Jaw and perioral tremor occurring while drinking from a glass, reduced while drinking with a straw; absent at rest and while speaking	NA	7 Hz rhythmic activity more prominent in the anterior digastric muscles compared with the masseter and medial pterygoid muscles; no co‐contraction of antagonist muscles	NA	No efficacy from propranolol, primidone or BoNT
Zhao et al, 2025	45	F	42	No	Jaw and perioral tremor occurring while drinking from a cup or a bottle and during the maintenance of certain mouth postures; absent at rest, while drinking with a straw and speaking.	No improvement with alcohol	NA	NA	BoNT to orolingual muscles with clinical benefit

*Note*: Age is reported in years.

Abbreviations: BoNT, botulinum toxin; EPSS, electrophysiological study of swallowing; F, female; M, male; NA, not available.

### Clinico‐Demographic Features

From a clinico‐demographic perspective, the published cases delineate a relatively homogeneous patient profile. Most cases occur in women, with onset typically in the sixth to seventh decade,^4^, ^6^, ^7^, ^8^, ^9^, ^11^, ^12^, ^14^ with only a single male case described.^5^ Symptom onset is typically insidious, with gradual evolution over time.^4^, ^5^, ^7^, ^8^, ^9^, ^10^, ^11^, ^12^, ^14^ However, an abrupt onset accompanied by irregular EMG bursts has been reported in one case, a pattern atypical for organic tremor and raising the possibility of a functional movement disorder.^6^ Family history is largely unremarkable, aside from an isolated report of a relative with a mild action‐induced tremor of uncertain significance.^4^ Brain MRI findings were unremarkable across reports.

### Phenomenology

A relevant subgroup of patients exhibited tremor strictly limited to the act of drinking from a cup or a bottle, with complete absence during speech, chewing or resting jaw position.^4^, ^5^, ^12^, ^14^ In some reports, tremor disappeared when patients drank through a straw,^4^, ^5^, ^6^, ^7^ whereas other cases described only a partial reduction,^8^, ^9^ pointing to a task‐specific but not strictly task‐exclusive phenomenon. Similarly, mild tremor improvement was reported upon touching the upper lip with the cup in one patient.^12^ Together, these observations are compatible with a sensory trick, underscoring the influence of posture and sensory input on tremor severity and supporting a possible dystonic mechanism in some cases.^15^

Other cases extended beyond strict task specificity, showing tremor in additional orofacial conditions.^6^, ^7^, ^10^, ^11^ For instance, tremor could also be triggered during speech or by maintaining a partially open mouth, while being absent in closed or widely opened positions of the jaw.^11^ In another report, tremor was also elicited during the maintenance of specific oral postures,^7^ reflecting a mixed task‐ and position‐dependent mechanism. These observations point to a phenomenology less strictly task‐specific and more consistent with posture‐dependent manifestations, potentially constituting variants of focal jaw dystonia.^16^ External aggravating and relieving factors were inconsistently reported; stress worsened symptoms,^6^, ^11^ whereas alcohol had no effect or modest benefit.^4^, ^11^

In addition to these phenomenological patterns, some cases showed temporal associations with antecedent factors. One patient developed drinking tremor shortly after gingival grafting, suggesting that sensory changes might have contributed to symptom onset.^12^ Another patient developed tremor nearly 20 years after extensive dental reconstruction,^7^ raising the possibility of long‐term sensory reorganization. In another case, the patient had played the trumpet for 8 years during high school,^4^ although a causal link appears unlikely given the long latency (approximately 30 years) and the fact that embouchure dystonia predominantly affects professional musicians rather than amateur players.^17^

### Neurophysiology

Electrophysiological findings are broadly consistent across published cases, with surface EMG typically demonstrating a regular 5–8 Hz rhythmic discharge compatible with focal orofacial tremor. The specific muscles expressing this activity vary across reports: some describe predominant digastric involvement, either alone^10^ or with masticatory muscles,^9^, ^11^ whereas others report activity mainly in the orbicularis oris,^4^, ^14^ with one report describing both orbicularis oris and digastric involvement.^5^ These patterns outline a phenomenologically uniform yet anatomically variable profile. Only one study reported irregular EMG bursts, a pattern difficult to align with true tremor.^6^

### Treatment

Oral tremor medications have shown no clear efficacy in reported cases (see Table 1). Given the focal nature of the tremor, BoNT represents the most appropriate treatment. When employed, injections targeted the digastric, masseter, and orbicularis oris muscles, with dosages tailored to the muscles involved.

## Discussion

The three cases presented here illustrate the clinical and phenomenological heterogeneity of drinking tremor, an uncommon orofacial tremor syndrome whose characterization remains incomplete and whose natural history remains poorly defined due to the lack of longitudinal data. Across our cases and prior reports, the marked female predominance and late‐life onset emerge as recurring features, suggesting a relatively homogeneous demographic profile, although no underlying mechanism has been established. Other reports describing jaw tremor associated with dystonia have shown similar clinical characteristics, although in those cases, clear dystonic signs were present or jaw tremor developed only years after dystonia onset.^18^ Conversely, cases of jaw tremor accompanied by parkinsonian signs have involved predominantly male patients aged 60–80 years.^19^ Beyond age‐ and sex‐related patterns, familial factors may also be relevant: both case 2 and case 3 reported a family history of late‐onset upper‐limb tremor, with additional chin involvement in the relative of case 3. A similar familial presentation has been described in at least one published report.^4^ The occurrence of tremor in affected relatives may suggest a possible hereditary background or familial predisposition, an aspect that warrants further investigation.

The pronounced clinical heterogeneity described in the literature reflects the variability observed in our series. A summary of the main clinical features across our cases is provided in Table 2. Differences emerged in the primary muscles involved and in the postures or motor task eliciting the tremor. In case 1, drinking triggered tremor of the jaw, perioral, and perinasal muscles; case 2 showed predominant involvement of the jaw and lips, while case 3 exhibited mainly jaw tremor with additional chin involvement. Jaw tremor occurred in all three patients, and in cases 2 and 3 it was also present at rest, suggesting a posture‐dependent component further exacerbated by precision tasks such as drinking. These observations highlight the interaction between task demands and orofacial posture, illustrating why task‐ and position‐specific tremor presentations often overlap rather than representing clearly distinct entities.^1^ As in previous reports, all three cases showed tremor suppression when drinking with a straw. Despite representing a precision task requiring coordinated orofacial activation, the maneuver may modulate sensorimotor integration and reduce tremor amplitude, acting as a sensory trick.^15^ In this regard, subtle dystonic postures involving the shoulder and head were observed in cases 2 and 3, suggesting a possible dystonic origin in these cases.^20^, ^21^ Kinematic analysis further supports a shared pathophysiological pattern across our cases. Despite clinical variability, all cases exhibited tremor frequencies between 6 and 7 Hz with comparable RMS amplitudes (Fig. 2). These values mirror the 5–8 Hz rhythmic activity reported in surface EMG studies, suggesting that different clinical expressions may arise from a common underlying tremor generator.^22^, ^23^

**TABLE 2:** Summary of orolingual motor tasks and additional neurological features in the three reported cases

	Case 1	Case 2	Case 3
Drinking	**++** (Jaw, perioral, perinasal muscles)	***++*** (Jaw, lips)	**++** (Jaw, chin)
Holding liquids in mouth	**−**	−	**−**
Mimicking drinking (empty glass)	**+**	**−**	**+**
Drinking with a straw	**−**	**−**	**−**
Eating	−	−	−
Face at rest	**−**	**+**	**−**
Mouth open	−	**++**	**+**
Whistle/smile	−	−	−
UULL rest tremor	**++** (Bilateral)	−	−
UULL action tremor	**+** (Postural and kinetic ‐Bilateral)	**+** (Postural ‐ Right side)	**+** (Kinetic ‐ Bilateral)
Finger tapping	Mild bradykesia and related features on the right side	−	Right mirror movements during left finger tapping
Gait	Reduced right arm swing and walking tremor	−	−
Other neurological signs	Subtle dystonic postures of left wrist, mild left shoulder elevation	Mild left shoulder elevation	Right laterocollis with left torticollis

*Note*: ++ marked; + mild; − absent.

Abbreviation: UULL, Upper limbs.

Case 1, however, exhibited bilateral rest tremor, mild bradykinesia and related features,^24^, ^25^ and reduced right arm swing with a walking tremor, a combination that could raise suspicion for Parkinson's disease. Yet the patient reported no prodromal symptoms, and the negative DaT‐SPECT strongly argues against nigrostriatal degeneration. Notably, similar parkinsonian signs, including rest tremor and mild bradykinesia, have also been described in dystonic tremor syndromes.^26^, ^27^ Longitudinal follow‐up is warranted to corroborate this diagnostic hypothesis and exclude an underlying nigrostriatal degeneration. This case underscores the diagnostic complexity of task‐specific tremor presentations and highlights the need for detailed phenomenological characterization, supported by neurophysiological data and longitudinal observation.^2^, ^20^, ^28^

Botulinum toxin treatment for orofacial drinking tremor can be directed toward different muscles depending on the individual pattern of involvement. In the literature, BoNT injections have been directed mainly to the masseter, digastric or orbicularis oris muscles, although occasional side effects such as dysphagia or impaired orobuccal function may occur.^29^, ^30^ Additional perioral targets, including the mentalis muscle, may also be considered in selected cases as part of a tailored injection strategy, given the low risk of significant adverse effects. An individualized BoNT treatment is warranted, balancing clinical benefit with tolerability and safety.

Drinking tremor shows marked phenomenological variability, ranging from predominantly task‐specific to more clearly posture‐dependent patterns, with boundaries between these forms often difficult to define. This condition occurs mainly in women in the sixth to seventh decade and may also present with tremor in additional body regions or subtle dystonic postures, sometimes accompanied by a possible sensory trick. Rather than defining a distinct nosological entity, our findings support the interpretation of orofacial drinking tremor as a clinically heterogeneous presentation, possibly reflecting a dystonic origin. Collectively, these features underscore the limitations of current tremor classifications and highlight the need for diagnostic approaches that more effectively integrate phenomenology with pathophysiological mechanisms.

## Author Roles

(1) Research project: A. Conception, B. Organization, C. Execution; (2) Statistical Analysis: A. Design, B. Execution, C. Review and Critique; (3) Manuscript: A. Writing of the first draft, B. Review and Critique:

D.B.: 1A, 1B, 1C, 3A

L.A.: 3B

S.A.: 3B

A.S.G.: 3B

S.C.: 3B

A.M.: 3B

M.D.R.: 3B

M.B.: 1A, 1B, 3B

## Disclosures

**Ethical Compliance Statement:** The authors confirm that approval of an institutional review board or ethics committee was not required for this case report. All procedures performed were in accordance with the ethical standards of our institution, and with the Helsinki Declaration. Written informed consent was obtained from the patient for the publication of his data and the online distribution of the related video material. We confirm that we have read the Journal's position on issues involved in ethical publication and affirm that this work is consistent with those guidelines.

**Funding Sources and Conflicts of Interest:** This work was supported by the Italian Ministry of Health (Current Research 2026). The authors declare that there are no conflicts of interest relevant to this work.

**Financial Disclosures for the Previous 12 Months:** The authors declare that they have no financial disclosures to declare.

## Financial Disclosures and Conflicts of Interest

Author disclosures are available in the Supporting Information.

## Supporting information


**Table S1.** Summary of botulinum toxin therapy and previous treatments in the three reported cases

**Data S1.** COI_disclosure.

## Data Availability

The data that support the findings of this study are available on request from the corresponding author. The data are not publicly available due to privacy or ethical restrictions.

## References

[1] Silverdale MA , Schneider SA , Bhatia KP , Lang AE . The spectrum of orolingual tremor—a proposed classification system. Mov Disord 2008;23(2):159–167. 10.1002/mds.21776. 17973324

[2] Bhatia KP , Bain P , Bajaj N , et al. Consensus Statement on the classification of tremors. From the task force on tremor of the International Parkinson and Movement Disorder Society. Mov Disord 2018;33(1):75–87. 10.1002/mds.27121. 29193359 PMC6530552

[3] Bain PG . Task‐specific tremor. Handb Clin Neurol 2011;100:711–718. doi:10.1016/B978-0-444-52014-2.00050-1. 21496617

[4] O'Gorman CM , Bower JH , Matsumoto JY , Kantarci OH , Kumar N . When drinking makes the tremor worse: a task‐specific orolingual tremor. Mov Disord Clin Pract 2014;1(3):237–239. 10.1002/mdc3.12041. 30363876 PMC6183252

[5] Stampanoni Bassi M , Casciato S , Gilio L , et al. Subclinical dysphagia in task‐specific mouth tremor triggered by drinking. Clin Neurophysiol 2019;130(8):1289–1291. 10.1016/j.clinph.2019.05.009. 31170653

[6] Benedek K , Biernat HB , Thomsen CE , Bakke M . Task‐specific drinking tremor. JMD 2023;16(1):98–100. 10.14802/jmd.22103. 36353802 PMC9978259

[7] Zhao CW , Song PC , Cheung JM , Kyle K . Orolingual drinking tremor treated with botulinum toxin. Neurology 2025;105(1):e213779, Jul. 10.1212/WNL.0000000000213779. 40472305

[8] Lau YH , Ong TL , Joseph JP , Mawardi AS . A rare case of late‐ onset task‐specific jaw tremor. J Clin Neurol 2023;19(4):416–418. 10.3988/jcn.2022.0202. 37417439 PMC10329931

[9] Rebelo Procaci V , De Azevedo LA , Barsottini OGP , Ferraz HB , Pedroso JL . Drinking Tremor. Mov Disord Clin Pract 2026;13(3):830–831. 10.1002/mdc3.70398. 41097989 PMC13042513

[10] Miles TS , Findley LJ , Rothwell JC . Electrophysiological observations on an unusual, task specific jaw tremor. J Neurol Neurosurg Psychiatry 1997;63(2):251–254. 10.1136/jnnp.63.2.251. 9285468 PMC2169678

[11] Tarsy D , Ro SI . Unusual position‐sensitive jaw tremor responsive to botulinum toxin. Mov Disord 2006;21(2):277–278. 10.1002/mds.20737. 16211606

[12] Carpentier A , Selfani K , Huot P . Task‐specific oro‐lingual tremor following gingival grafting surgery. J Neurol Sci 2016;367:24–25. 10.1016/j.jns.2016.05.043. 27423558

[13] Birreci D , Angelini L , Paparella G , et al. Pathophysiological role of primary motor cortex in essential tremor. Mov Disord 2025;40(8):1648–1660. 10.1002/mds.30197. 40243615 PMC12371654

[14] Macerollo A , Meppelink AM , Teodoro T , Ricciardi L , Cordivari C , Edwards MJ . Isolated task‐specific lip tremor. Parkinsonism Relat Disord 2016;29:138–139. 10.1016/j.parkreldis.2016.04.019. 27118488

[15] Schramm A , Classen J , Reiners K , Naumann M . Characteristics of sensory trick‐like manoeuvres in jaw‐opening dystonia. Mov Disord 2007;22(3):430–433. 10.1002/mds.21354. 17226856

[16] Balal M , Demirkiran M . Oromandibular dystonia: clinical and demographic data from eight‐two patients. Tremor Other Hyperkinet Mov (N Y) 2023;13:3. 10.5334/tohm.730. 36789171 PMC9896995

[17] Frucht SJ . Embouchure dystonia‐‐portrait of a task‐specific cranial dystonia. Mov Disord 2009;24(12):1752–1762. 10.1002/mds.22550. 19562760

[18] Schneider SA , Bhatia KP . The entity of jaw tremor and dystonia. Mov Disord 2007;22(10):1491–1495. 10.1002/mds.21531. 17469206

[19] Baizabal‐Carvallo JF , Alonso‐Juarez M , Fekete R . Lip and jaw tremor in Parkinson's disease. Tremor Other Hyperkinet Mov (N Y) 2025;15:13. 10.5334/tohm.1001. 40248112 PMC12005137

[20] Albanese A , Bhatia KP , Fung VSC , et al. Definition and classification of dystonia. Mov Disord 2025;40(7):1248–1259. 10.1002/mds.30220. 40326714 PMC12273609

[21] Fearon C , Espay AJ , Lang AE , et al. Soft signs in movement disorders: friends or foes? J Neurol Neurosurg Psychiatry 2019;90(8):961–962. 10.1136/jnnp-2018-318455. 30409889

[22] Goede LL , al‐Fatly B , Li N , et al. Convergent mapping of a tremor treatment network. Nat Commun 2025;16(1):4772. 10.1038/s41467-025-60089-6. 40404653 PMC12098757

[23] Buijink AWG , Van Rootselaar A‐F , Helmich RC . Connecting tremors – a circuits perspective. Curr Opin Neurol 2022;35(4):518–524. 10.1097/WCO.0000000000001071. 35788547

[24] Paparella G , de Riggi M , Cannavacciuolo A , et al. Analyzing the “bradykinesia complex” in Parkinson's disease. Mov Disord 2025;41:143–155. 10.1002/mds.70082. 41104587 PMC12882039

[25] Bologna M , Espay AJ , Fasano A , Paparella G , Hallett M , Berardelli A . Redefining Bradykinesia. Mov Disord 2023;38(4):551–557. 10.1002/mds.29362. 36847357 PMC10387192

[26] Schneider SA , Edwards MJ , Mir P , et al. Patients with adult‐onset dystonic tremor resembling parkinsonian tremor have scans without evidence of dopaminergic deficit (SWEDDs). Mov Disord 2007;22(15):2210–2215. 10.1002/mds.21685. 17712858

[27] Angelini L , Paparella G , Schwingenschuh P , Helmich RCG , Bologna M . Long term (TEN YEARS) follow‐up in a trembling patient with parkinsonian signs without dopaminergic denervation. Clin Parkinson Relat Disord 2025;13:100371. 10.1016/j.prdoa.2025.100371. PMC12311531 40746845

[28] Postuma RB , Berg D , Stern M , et al. MDS clinical diagnostic criteria for Parkinson's disease: MDS‐PD clinical diagnostic criteria. Mov Disord 2015;30(12):1591–1601. 10.1002/mds.26424. 26474316

[29] Ramirez‐Castaneda J , Jankovic J , Comella C , Dashtipour K , Fernandez HH , Mari Z . Diffusion, spread, and migration of botulinum toxin. Mov Disord 2013;28(13):1775–1783. 10.1002/mds.25582. 23868503

[30] ‘Use of Botulinum Toxin in Orofacial Clinical Practice’. Accessed: Nov. 29; 2025. [Online]. Available: https://www.mdpi.com/2072-6651/12/2/112.

