# Supplementary material for: Orofacial Drinking Tremor: A Case Series and Literature Review
Source: Mov Disord Clin Pract. 2026 Apr 10:10.1002/mdc3.70621. Online ahead of print. doi: 10.1002/mdc3.70621 (PMC13339577; doi:10.1002/mdc3.70621)
Supplement: Supplementary file 4 — Table S1. Summary of botulinum toxin therapy and previous treatments in the three reported cases [file MDC3-9999-0-s001.docx]

**Supplementary table 1. Summary of botulinum toxin therapy and previous treatments in the three reported cases**

| **Cases** | **BoNT formulation** | **Dose and injected muscles** | **Previous BoNT attempts** | **Duration of BoNT therapy** | **Previous treatments** |
| --- | --- | --- | --- | --- | --- |
| Case 1 | Onabotulinum toxin type A | 5 UI in each mentalis muscle, 5 UI in each masseter muscle | 5 UI in each mentalis muscle | 9 months | Pramipexole extended release 0.52 mg/day (no benefit); propranolol 20 mg/day (stopped for bradycardia); clonazepam 0.6 mg/day (stopped for daytime sleepiness) |
| Case 2 | Incobotulinum toxin type A | 7.5 UI in each mentalis muscle | NA | 3 years | Propranolol 40 mg/day + clonazepam 0.5 mg/day (minimal benefit) |
| Case 3 | Onabotulinum toxin type A | 5 UI in each mentalis muscle, 5 UI in each masseter muscle | 5 UI in each mentalis muscle | 2 years | No previous treatment for tremor |

Abbreviations: BoNT, botulinum toxin, NA, not applicable
